# A New Record of *Pogonatum tahitense* (Polytrichaceae) from Tibet, China: Taxonomic Description, Range Expansion, and Biogeographic History

**DOI:** 10.3390/plants13060846

**Published:** 2024-03-15

**Authors:** Yu Sun, Xiaotong Song, Chunfa Chen, Shuang Li, Jiqi Gu, Xiaoming Shao

**Affiliations:** 1 Lushan Botanical Garden, Chinese Academy of Sciences, No. 9, Zhiqing Rd., Jiujiang 332900, China; suny@lsbg.cn (Y.S.); chencf@lsbg.cn (C.C.); lis@lsbg.cn (S.L.); 2 College of Life Science, Nanchang University, Nanchang 330031, China; 3 College of Resources and Environmental Sciences, China Agricultural University, Beijing 100193, China; songxiaot_1@163.com (X.S.); gujq@mail.bnu.edu.cn (J.G.); 4 Beijing Key Laboratory of Biodiversity and Organic Farming, China Agricultural University, Beijing 100193, China; 5 State Key Laboratory of Earth Surface Processes and Resource Ecology, College of Life Sciences, Beijing Normal University, Beijing 100875, China; 6 Resources & Environment College, Tibet Agriculture & Animal Husbandry University, Nyingchi 860000, China

**Keywords:** *Pogonatum tahitense*, Polytrichaceae, moss, taxonomy, phylogeny, distribution, biogeographic history

## Abstract

The genus *Pogonatum* stands out as the most diverse within the family Polytrichaceae, encompassing over 50 species. *Pogonatum tahitense* has been recorded across various Pacific regions, including Hawaii in the United States and Tahiti in French Polynesia, as well as in Asia, such as in Taiwan in China, Java in Indonesia, and Sabah in Malaysia. In the current study, a specimen collected in Tibet, China, is described, confirming its taxonomic classification as *P. tahitense* through a comprehensive analysis integrating morphological evidence and molecular study based on sequences from the plastid (*rbcL*, *rps4*, *trnL-F*), mitochondrial (*nad5*), and nuclear (*ITS2*) regions. This documentation represents the first record of the species within mainland China. A time-calibrated, molecular-based phylogenetic analysis was conducted, employing various approaches for ancestral range inference. The findings suggest that *P. tahitense* originated during the Pleistocene epoch, approximately 1.8 mya, in Tibet, China.

## 1. Introduction

The family Polytrichaceae Schwägr. [1] encompasses approximately 200 species and comprises 17 extant genera [2]. *Pogonatum* P. Beauv. [3] stands out as the most diverse genus within the family, containing over 50 species characterized by distinct morphological features of sporophytes, including a lack of stomata, deeply pigmented peristome, and a mammillose exothecium [2,4]. Previous phylogenetic studies consistently posited *Pogonatum* as monophyletic, positioned as the sister genus to *Polytrichum* Hedw. [5]; however, the subgeneric classification remains unequivocally unresolved [2,6,7].

*Pogonatum tahitense* Besch. [8], initially nominated by Schimper based on a specimen originating from Tahiti, French Polynesia, stands apart from other congeners due to its small size, narrow leaves, and irregularly crenate and geminate apical cells of the lamellae [4,9,10]. This species occurs in open habitats, growing on exposed soil banks, as well as on clay and rocks, within the altitudinal range of 300 to 1400 m [4,9,10]. Geographically, *P. tahitense* has been documented across various Pacific islands, including Hawaii in the United States and Tahiti in French Polynesia, as well as in Southeast Asian islands, such as Taiwan in China, Java in Indonesia, and Sabah in Malaysia [4,9,10]. 

The disjunctive distribution pattern between the Himalayas and Taiwan has been observed in species within this genus, such as *P. microstomum* and *P. shevokii*, although the biogeographical history underlying this pattern remains unexplored [4,11]. This pattern of disjunction aligns with the biogeographical narratives derived from the phylogenetic and molecular dating studies of vascular plants, which have posited the Himalayas as an origin point for the genera *Theligonum*, *Kelloggia*, *Triplostegia*, and *Prinsepia* [12,13,14].

The present study describes a specimen collected in Tibet, China, affirming its taxonomic identity as *P. tahitense* through comprehensive morphological and molecular analyses. This marks the first documentation of the species within mainland China. In elucidating the biogeographic history of *P. tahitense*, we reconstruct its divergence time and ancestral distribution patterns.

## 2. Results

### 2.1. Morphological Study

Specimens examined: China, Tibet, Nyingchi City, Bayi District, the eastern slopes of Shergyla Mountain, next to National Highway 318, and along the Lulang River, 29.867652° N, 94.775124° E, elevation 2939 m, in mixed conifer forests, on soil, 3 August 2020, by Xiaoming Shao, Jiqi Gu, and Ling Liu, 20200803SGL062 (BAU). 

**Figure 1:**
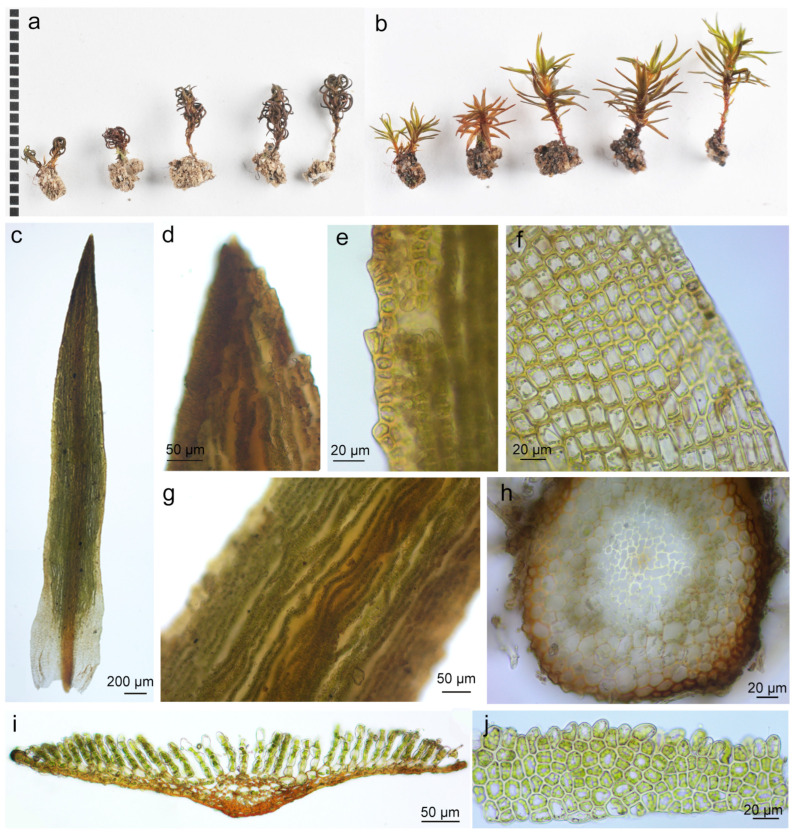
*Pogonatum tahitense* Besch. (**a**) Habits (dry); (**b**) habits (wet); (**c**) leaf; (**d**) leaf apex; (**e**) upper leaf cells and margin; (**f**) basal leaf cells; (**g**) lamellae; (**h**) stem cross-section; (**i**) leaf middle cross-section; (**j**) lamella in side view.

Description: Plants dwarfed, green to brownish red, loosely gregarious. Stems erect, single, ca.4–10 mm high, 0.4–0.8 mm wide with leaves. Leaves 3–4 mm long and 0.5–0.6 mm broad (mid-leaf), spirally arranged, plane and erect-spreading when moist, contorted and incurved when dry, narrow lanceolate, apex acuminate; sheath short-ovate, not hyaline-margined. Costa ending in the tip and weakly toothed abaxially near the tip. Leaf margins single-celled, with small and rounded teeth, teeth vanishing near the middle of the leaf. Lamellae arranged in 30–35 ranks, slightly undulate, 2–5 cells high, apical cells of lamellae single or in pairs, smooth, irregularly crenate on side view. Leaf abaxial cells irregularly rounded- quadrangular, basal leaf cells rectangular. Sporophytes not found (Figure 1). 

### 2.2. Phylogenetic Relationship

Optimal models for analyzing the *rbcL*, *rps4*, *trnL-F*, *nad5*, and *ITS2* regions were determined via ModelTest-NG v0.1.7 by employing the corrected Akaike information criterion (AICc). The outcomes indicated that the GTR + I + G was most suitable for the *rbcL* and *rps4* regions, whereas the HKY + I + G was selected for the *trnL-F*, *nad5*, and *ITS2* regions. These models were subsequently applied to their respective partitions in Bayesian analyses. Due to raxmlGUI’s limitation in accommodating diverse models for distinct partitions, the HKY + I + G model was uniformly applied in the maximum likelihood analysis. 

**Figure 2:**
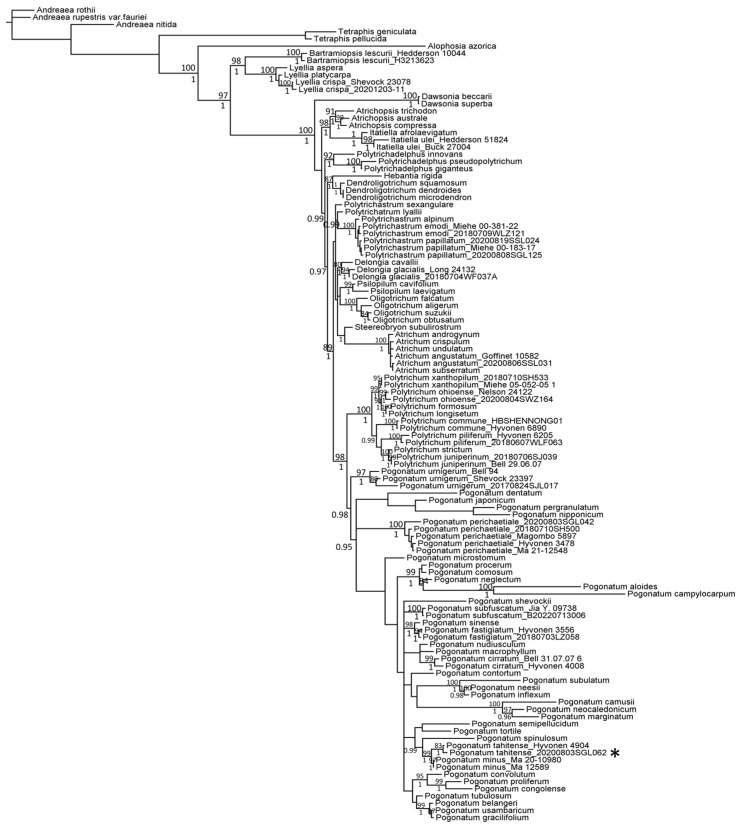
The phylogenetic tree of Polytrichaceae was established through the 50% majority-rule consensus tree obtained from a Bayesian analysis conducted using MrBayes v3.2.7 based on *rbcL*, *rps4*, *trnL-F*, *nad5*, and *ITS2* regions. Indications of Bayesian posterior probabilities surpassing 0.95 are annotated below the branches, and bootstrap values larger than 80% from maximum likelihood analysis are shown above the branches. Asterisk indicates the specimen collected from Tibet, China.

The phylogenetic reconstructions from individual regions were characterized by unresolved topologies with low support values. The 50% majority-rule consensus trees for each region and the organellar dataset are available in the Supplementary Material (Figures S1–S6). Consistency and robust support across the individual topologies were observed in a limited number of clades, notably including *Bartramiopsis*, *Lyellia*, *Dawsonia*, and *Atrichum*. The concatenated analysis of organellar regions yielded significantly improved phylogenetic resolution, with all Polytrichaceae genera, except *Polytrichastrum* and *Pogonatum*, being delineated as monophyletic entities (Figure S6). Despite the ITS2 nuclear region dataset containing fewer samples relative to the organellar datasets, it was included in this study due to its coverage of *P. tahitense* sample from Hawaii (Figure S5); as a result, the combined organellar and nuclear datasets produced a phylogenetic tree with enhanced topology and support values (Figure 2, Figures S7 and S8). 

The congruence between the topology obtained from both the Bayesian and maximum likelihood analyses is evident (Figure 2, Figures S7 and S8). Notably, Polytrichaceae emerged as a monophyletic entity, with all genera forming robust, well-supported monophyletic clades, except for *Polytrichastrum* and *Pogonatum*. Specifically, *Polytrichastrum sexangulare* was located at a distinct branch separate from the main *Polytrichastrum* clade in both Bayesian and maximum likelihood analyses. *Pogonatum*, on the other hand, demonstrated monophyletic unity in the Bayesian analysis, but was paraphyletic in the maximum likelihood analysis. The samples of *Pogonatum* resolved in one clade with a low bootstrap value on 0.46 in the maximum likelihood analysis (Figure 2, Figures S7 and S8). Furthermore, the subclades within *Pogonatum* showed weak support across analyses, as indicated by low Bayesian posterior probabilities and bootstrap values. The specimen from Tibet exhibited a well-supported placement within a clade that included *P. minus* and *P. tahitense* in all analyses of the individual and combined dataset (Figures S3 and S5–S8). Intriguingly, the Tibetan specimen and *P. tahitense* sample from Hawaii were resolved within a single clade, with a high posterior probability of 1 and a bootstrap value of 83% (Figure 2, Figures S7 and S8). 

### 2.3. Divergence Time and Biogeographic History

**Figure 3:**
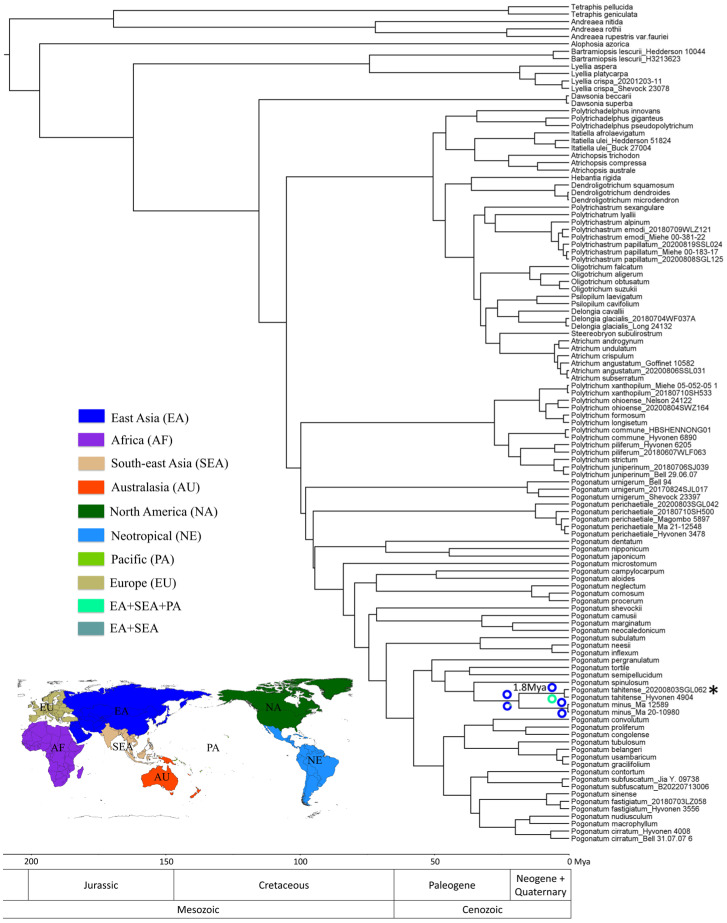
The phylogenetic dated tree of Polytrichaceae was established through the derivation of a maximum clade credibility tree. This tree was constructed via Bayesian inference analysis performed in the BEAST v1.10.4 software, utilizing absolute substitution rates as a basis. Asterisk indicates the specimen collected from Tibet, China. The divergence time of *P. tahitense* is given above the branch. The ancestral biogeographic areas for Polytrichaceae were reconstructed utilizing the statistical dispersal–vicariance analysis (S-DIVA). Nodes in the *P. tahitense* and *P. minus* clades are represented by colored circles denoting the ancestral area, and portions of different colors indicate the probability of suggested ancestors. Colored circles above the branch were reconstructed utilizing S-DIVA analysis, whereas the results from ancestral reconstruction by DEC were shown below the branch. The delineation of eight biogeographic areas was shown in the colored world map. The geologic time scale is presented at the bottom for reference.

The results obtained from the analysis using absolute substitution rates of molecular evolution (Figure S9) aligned with those derived from fossil calibrations (Figure S10). According to the estimated divergence dates using absolute substitution rates, the initial diversification event within Polytrichaceae occurred around the Lower Jurassic period, approximately 197 mya, with a confidence interval ranging from 173 to 223 mya (Figure S9). In the context of biogeographical scenarios, the S-DIVA analysis proposed Asia and Europe as the ancestor area for Polytrichaceae (Figure S11). According to the findings from the divergence time analysis and ancestral area reconstruction, the ancestor of *P. tahitense* originated around 1.8 million years ago in Tibet. Subsequently, it underwent dispersal to Taiwan, as well as the islands in Southeast Asia (Java and Borneo) and the Pacific region (Hawaii and Tahiti) (Figure 3 and Figure S11). The outcomes of the *P. tahitense* clade derived from the S-DIVA and DEC analyses are provided in Figure 3. For the detailed results stemming from both analyses, references can be obtained from Figures S11 and S12.

## 3. Discussion

### 3.1. Phylogenetic Relationships

Topological incongruence was evident in the 50% majority-rule consensus trees derived from analyses of individual regions (Figures S1–S5). Consistent congruence and robust support were observed in a limited number of clades across the topologies of individual regions, notably including *Bartramiopsis*, *Lyellia*, *Dawsonia*, and *Atrichum*. In the independent analyses of the *rbcL* region, *Pogonatum aloides* and *Pogonatum campylocarpum* were resolved alongside the outgroup species *Tetraphis geniculata* and *T. pellucida*, whereas analyses of the *rps4* region associated them with *Alophosia azorica* (Figures S1 and S2). This finding aligns with previous research, where *P. aloides* and *P. campylocarpum* were grouped with *A. azorica* in a maximum likelihood analysis utilizing *18S* loci. These aberrant phylogenetic associations are attributed to the phenomenon of long-branch attraction [15,16]. Our current study validates that integrating data from multiple regions ameliorates the issues associated with long-branch attraction, thereby enhancing the phylogenetic analysis in terms of topology and support values (Figure 2, Figures S7 and S8). 

The congruence in topologies yielded by both Bayesian and maximum likelihood analyses was remarkable. The *Polytrichaceae* family was unequivocally identified as a monophyletic group, with all constituent genera forming robust, well-supported monophyletic clades, with the exception of *Polytrichastrum* and *Pogonatum*. Specifically, *Polytrichastrum sexangulare* was consistently placed on a distinct branch, divergent from the primary *Polytrichastrum* clade, in both analytical approaches. Previous research had similarly found *P. sexangulare* to be poorly resolved with the *Polytrichastrum* clade [7,17,18], suggesting that *P. sexangulare* may represent one previously undescribed genus [2]. 

### 3.2. Pogonatum Tahitense and Its Related Species

The specimen of *P. tahitense* collected from Tibet exhibits notable similarities to the counterparts from Hawaii and Taiwan, sharing almost all morphological features [4,9]. The only discernible difference lies in the frequency of geminations observed in the apical cells, which are abundant in specimens from Hawaii and Taiwan [4,9] while being relatively rare in the specimen from Tibet. While Pacific and Southeast Asian Island specimens typically thrive in open sites within the altitude range of 300 to 1400 m [4,10], the Tibetan collection’s habitat closely resembles these islands but at a higher altitude of 2939 m. The observed distinctions between the Tibetan specimen and those from other regions are considered insufficient to warrant the designation of the Tibetan sample as a new taxon. Rather, we attribute these differences to local environmental adaptations, a phenomenon commonly observed in various moss species in response to environmental variations. Noteworthy examples include the varied length of sporophytic setae and the size of spores in *Pyrrhobryum spiniforme* and *Neckeropsis undulata* corresponding to lowland and highland habitats, as well as the morphological variations in *Bryum argentem* influenced by diverse climate and soil factors [19,20]. Consequently, we classify the Tibetan specimen as conspecific with *P. tahitense*, supported by both morphological and molecular analyses. 

Previous studies proposed a close relationship between *P. tahitense* and the African species *P. gracilifolium*, *P. tubulosum* from Indonesia, Papua New Guinea, and Australia, as well as *P. minus* endemic to Yunnan, China [4,9]. Shared features among these species include small, rounded marginal teeth and an excurrent, wide costa [4]. However, each species possesses distinctive traits: *P. gracilifolium* exhibits numerous dorsal teeth on the excurrent costa, contorted leaves when dry, and irregular apical cells of the lamellae with some geminations [4]. *P. tubulosum* displays smooth apical cells of the lamellae, somewhat irregularly crenate on the side view, and at least some retuse apical cells [4]. *P. minus* has contorted leaves when dry with a smooth costa on the dorsal side [4]. *P. tahitense* is characterized by weakly toothed abaxial regions of costa near the tip and round or oblong apical cells of lamellae [4] (Figure 1). Our phylogenetic analyses supported *P. minus* as the sister species of *P. tahitense*, whereas *P. gracilifolium* and *P. tubulosum* clustered in a clade predominantly composed of species distributed in Africa (Figure 2). Interestingly, *P. spinulosum* emerged as the sister species of *P. tahitense* and *P. minus*, collectively forming a well-supported clade with a 0.99 posterior probability in the Bayesian analysis (Figure 2 and Figure S7). While all three species share a diminutive size and an Asian distribution, *P. spinulosum* possesses distinct features, such as a persistent protonema, leaves appressed on stems, and the absence of lamellae, rendering its phylogenetic position uncertain [4]. Notably, many *Pogonatum* species remained unresolved with low posterior probability in our Bayesian analysis, consistent with previous studies that exhibited similar topologies [2,6,7]. It is crucial to acknowledge that previous analyses primarily utilized DNA fragments for elucidating the phylogenetic relationships within *Pogonatum* [2,6,7]. Thus, a comprehensive phylogenomic analysis of this genus is imperative to resolve its intricate phylogenetic relationships.

### 3.3. Biogeographic History of Distribution Patterns

The biogeographic history inferred using both the DEC and S-DIVA analyses exhibited variations in delineating ancestral areas. The DEC analysis indicated a broader range of ancestral areas compared to the S-DIVA analysis. For instance, the ancestral area inferred for Polytrichaceae encompassed all continents, while the ancestral range for *P. tahitense* was identified to include Asia, Southeast Asia, and the Pacific region (Figure 3, Figures S11 and S12). Notably, the DEC model has been observed to exhibit a tendency toward overestimating extinction frequencies, imposing constraints on dispersal, and inferring ancestral ranges that extend beyond the current distribution of extant species [21]. Therefore, we posit that the paleobiogeographical scenarios inferred through S-DIVA analysis are more plausible and align better with the empirical data than those derived from the DEC model. 

Our dataset encompasses 77 species from the Polytrichaceae family, which includes 31 species classified under the genus *Pogonatum*. The geographic distribution of our sampling effort is as follows: Asia is represented by 48 species of Polytrichaceae, including 22 species of *Pogonatum*; Southeast Asia is represented by 35 species of Polytrichaceae and 19 species of *Pogonatum*; Africa is represented by 17 species of Polytrichaceae and 8 species of *Pogonatum*; Europe is represented by 18 species of Polytrichaceae and 3 species of *Pogonatum*; North America is represented by 24 species of Polytrichaceae and 3 species of *Pogonatum*; the Neotropical region is represented by 22 species of Polytrichaceae and 2 species of *Pogonatum*; Australasia is represented by 20 species of Polytrichaceae and 7 species of *Pogonatum*; the Pacific is represented by 8 species of Polytrichaceae and 2 species of *Pogonatum*. This sampling effort covers less than 50% of the extant species within the Polytrichaceae family, with notable gaps in the Neotropical and African regions [2]. Consequently, these omissions may potentially introduce bias in the reconstruction of ancestral areas, particularly affecting the inference of dispersal events within specific genera due to the absence of key species. However, the impact of sampling bias on the reconstruction of deeper phylogenetic nodes may be mitigated, as our dataset includes representatives from all 17 extant genera within the family, encompassing the primary diversity regions of each genus [2]. Our analysis revealed that Polytrichaceae originated in Asia and Europe during the Lower Jurassic period (Figure S11). The presence of the European component is notably influenced by *Alophosia azorica*, identified as the earliest diverging species and endemic to Azores Archipelago. During the Late Miocene, when the Azores Archipelago started to surface above sea level [22,23,24], it is hypothesized that the initial geographical range of Polytrichaceae was predominantly situated in Asia. The migration of *A. azorica* to the Azores, we suggest, transpired after the formation of the islands [22,23,24]. Previous research suggested that the earliest diverging genera, including *Alophosia*, *Bartramiopsis*, and *Lyellia*, which predominantly inhabit the Azores, Asia, and North America, are relict [2]. Drawing on the outcomes of our divergence time estimation and ancestral area reconstruction analyses, we propose that the Polytrichaceae family likely originated in Laurasia and subsequently underwent an early radiation event during the Cretaceous period. This radiation event facilitated the dispersal of *Dawsonia* to Australasia, while the genera *Dendroligotrichum*, *Hebantia*, *Polytrichadelphus*, *Atrichopsis*, and *Itatiella* dispersed to the Neotropics (Figures S9–S12). Given the acknowledged sampling bias, coupled with the considerable phylogenetic uncertainty highlighted by the pronounced disparities between the Bayesian analysis and the dated ultrametric BEAST tree, as well as the low support values for deeper nodes, our discussion on the divergence and biogeographic history within Polytrichaceae is focused exclusively on *P. tahitense*, thereby avoiding speculative conclusions about other species and genera within the family.

**Figure 4:**
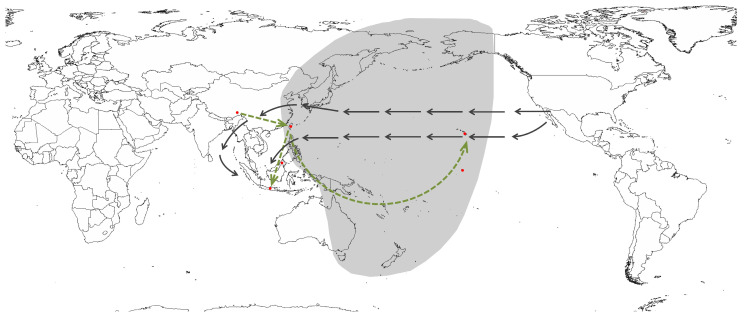
Map of dispersal routes of *P. tahitense*. Red dots show the current distribution of *P. tahitense*. Green dashed line arrows depict the proposed dispersal routes identified in this study. Black arrows indicate the tropical easterlies in the Asian–Pacific region, while the gray shadow area represents the West Pacific Flyway.

The current distribution pattern of *P. tahitense* exhibits a disjunct pattern, occurring in Tibet and Taiwan in China, as well as islands in Southeast Asia and the Pacific [4,9,10]. According to our ancestor area reconstruction, *P. tahitense* was present in Tibet around 1.8 mya, subsequently dispersed to Taiwan, followed by further dispersals to Southeast Asian islands (Java and Borneo) and Pacific islands (Hawaii and Tahiti). As all relevant geographic events, including the uplift of the Himalaya and the emergence of those islands, had concluded by 1.8 mya [25,26,27,28,29,30,31,32], the disjunct distribution pattern of *P. tahitense* is attributed to dispersal rather than vicariance. The present distribution between Tibet and Taiwan is hypothesized to be the result of eastward dispersal, possibly facilitated during the Pleistocene glaciation when *P. tahitense* in Tibet moved to lower altitudes and eventually reached Taiwan through the exposed Fujian–Taiwan land bridge (Figure 4). The species likely retreated to mountainous areas as temperatures rose during the post-glacial epoch. However, due to the absence of suitable high mountain environments in Southeast China [33], the species became extinct in that region, leading to the current disjunct distribution between Tibet and Taiwan (Figure 4). This disjunct pattern between Taiwan and the Qinghai–Tibet Plateau is a recurring phenomenon observed in various bryophyte and plant species [11,14,34,35,36,37].

The southwestward dispersal from Taiwan to Sabah and Java is postulated to be driven by the tropical easterlies (Figure 4). The small size of *Pogonatum* spores (mostly less than 20 μm) makes them light enough to be wind-dispersed over thousands of kilometers [38]. Conversely, the eastward dispersal from Taiwan to Hawaii and Tahiti is unlikely to be wind-driven, because of the impracticality of dispersing eastward against the prevailing westward tropical easterlies; instead, it is presumably facilitated by bird migration (Figure 4). Research by Price and Wagner reported that Southeast Asia serves as the second-largest source region for the origins of the Hawaiian flora [39]. Lineages originating in Southeast Asia primarily reached the Pacific Islands through bird migration along the West Pacific Flyway [39]. For instance, investigations have revealed that fledglings of the Hawaiian petrel (*Pterodroma sandwichensis*) traverse southwestward to Melanesia and then turn northwest, reaching the Philippines and Taiwan, in China, in winter, ultimately returning to Hawaii for breeding in summer [40]. Furthermore, certain avian species, such as the ruddy turnstone (*Arenaria interpres*), red knot (*Calidris canutus*), and curlew sandpiper (*Calidris ferruginea*), undertake migratory journeys from China to New Zealand [41]. Others, including the Pacific golden-plover (*Pluvialis fulva*), wandering tattler (*Tringa incana*), and bristle-thighed curlew (*Numenius tahitiensis*), migrate between New Zealand and Alaska, USA [42]. *P. tahitense* is, therefore, likely dispersed to Tahiti and Hawaii indirectly via Pacific Islands (Figure 4).

For a more comprehensive understanding of the mechanisms underlying the distribution pattern and dispersal modes, future sampling efforts and genetic analyses at the population level of *P. tahitense* are essential. The discovery of new records of *P. tahitense* in mountainous areas of Southeast China and on additional Pacific Islands during future sampling efforts could provide valuable insights.

## 4. Materials and Methods

### 4.1. Taxon Sampling

The specimen of *P. tahitense* (20200803SGL062) from Tibet was collected in 2020 by Xiaoming Shao, Jiqi Gu and Ling Liu in Nyingchi, Tibet, China. To investigate its phylogenetic relationship, a DNA sequence dataset of 113 samples was compiled, including 108 samples from Polytrichaceae and 5 samples from Andreaeaceae and Tetraphidaceae as outgroups. The dataset covered three chloroplast DNA regions (*rbcL*, *rps4*, *trnL-F*), one mitochondrial region (*nad5*), and one nuclear region (*ITS2*). New sequences for the 5 regions were generated from 21 samples collected in Tibet, Hubei, and Yunnan in China, while the remaining sequences for the other 92 samples were obtained from GenBank. The voucher specimens utilized for sequencing in this study have been deposited in two herbaria: the China Agricultural University (BAU) and the Kunming Institute of Botany, Chinese Academy of Sciences (KUN). Details encompassing species names, voucher information, and GenBank accession numbers of the dataset can be found in Table S1.

### 4.2. Morphological Study

The original descriptions and illustrations of the type specimen of *P. tahitense* were studied, along with the previous publications on *P. tahitense* [4,8,9]. Microscopic photography was conducted using a digital camera attached to the microscope (AXIO Lab. A1, ZEISS, Oberkochen, Germany). All species names used in this study adhere to the nomenclature provided by TROPICOS, recognized as an up-to-date and reliable nomenclature database [43].

### 4.3. DNA Isolation, PCR, and Sequencing

The DNA extraction procedure involved using the SDS DNA extraction method [44] on dried herbarium material. PCR (polymerase chain reaction) was conducted using specific primer combinations for different target regions: *rbcL* (rbcLF and rbcLR) [45], *rps4* (trnas and rps5) [46], *trnL-F* (trnC and trnF) [47], *nad5* (nad5K and nad5Li) [48], and *ITS2* (5.8SR and LS4R) [49]. Taq DNA polymerase from Sangon Biotech facilitated the amplification, following a PCR program comprising an initial denaturation at 94 °C for 4 min, 35 cycles at 94 °C for 30 s, 48 °C for 30 s, and 65 °C for 2 min, concluding with a final extension of 10 min at 72 °C. Subsequently, the PCR products underwent purification and sequencing via the shotgun sequencing method at Beijing Tsingke Biotech Co., Ltd. (Beijing, China). Sequencing utilized the same primers for both forward and reverse DNA sequences. Assemblies of the sequences for each molecular region were accomplished using BioEdit v7.0.4.1 software [50]. Alignment of the initial sequences for the five molecular regions was executed using MAFFT v7.520 [51] in Jalview v2.11.3.2 [52,53,54], followed by manual adjustments to remove low-quality ends.

### 4.4. Phylogenetic Analyses

We employed the ModelTest-NG v0.1.7 [55,56] implemented in raxmlGUI v2.0 [57,58] to ascertain the most appropriate model for analyzing the *rbcL*, *rps4*, *trnL-F*, *nad5*, and *ITS2* regions, in addition to the concatenated datasets of organellar regions. To examine the incongruence across these various regions, Bayesian inference analyses were performed on each individual region as well as on the concatenated datasets of chloroplast and mitochondrial regions. Bayesian analyses were conducted by using MrBayes v3.2.7 [59,60,61] on the Cyberinfrastructure for Phylogenetic Research Science (CIPRES) Gateway v.3.3 platform [62]. Four independent runs were executed, encompassing ten million generations each, with four chains, and tree and parameter sampling carried out every hundred generations. Parameters were unlinked to allow independent variation across compartments. The analyses adhered to the optimal model identified for all five partitions within each dataset, as determined by the estimations provided by ModelTest-NG v0.1.7 [55,56]. The first 25% of the samples, deemed as burn-in due to their instability, were excluded, with the subsequent samples being aggregated to construct a 50% majority-rule consensus tree.

For the concatenated dataset encompassing the five regions, maximum likelihood (ML) analyses were executed utilizing RAxML v8.2.12 [63,64], interfaced through raxmlGUI v2.0 [57,58]. The analysis was conducted under the HKY + G + I model, employing the ‘ML+ thorough bootstrap’ feature within raxmlGUI v2.0 (corresponding to the option ‘-b’ followed by an ML search), with 50 iterations and 1000 bootstrap replications to ensure robust statistical support.

### 4.5. Divergence Time Estimates

The estimation of divergence times was conducted within a Bayesian framework utilizing BEAST v1.10.4 [65] and incorporating previously established substitution rates of plastid, mitochondrial, and nuclear DNA. Prior studies have reported an average absolute substitution rate of 5.0 × 10^−4^ substitutions per site per mya in cpDNA across diverse algae and land plants [66,67]. In line with this, we adopted a normal distribution, specifying a mean of 5.0 × 10^−4^ and a standard deviation of 10^−4^ substitutions per site per mya as priors for the absolute rates of evolution in the *rbcL*, *rps4*, and *trnL-F* regions. Gaut’s findings suggested an average absolute substitution rate of 1.09 × 10^−4^ substitutions per site per mya in the mitochondrial genome of land plants [68]. Accordingly, we employed a normal distribution with a mean of 1.09 × 10^−4^ and a standard deviation of 0.5 × 10^−4^ substitutions per site per mya for the rates of evolution in the *nad5* region. Considering sequence divergence estimates in the *ITS* region ranging from 0.008 to 0.02 per mya in green algae and land plants [69], we implemented a normal distribution with a mean of 0.014 and a standard deviation of 0.005 substitutions per site per Myr as a prior for the absolute *ITS2* rate of evolution. To mitigate potential effects stemming from substitution rate heterogeneity and uncertainties in fossil data [70], an uncorrelated lognormal relaxed clock model was employed. The substitution model GTR + G + I was employed with gamma categories set to 6, and a Yule tree prior was selected as the tree model. Markov chain Monte Carlo (MCMC) chains were executed for 10 million generations, with parameters and trees logged at intervals of 1000 generations. Tracer v1.7 [71] was utilized for parameter validation, and TreeAnnotator v1.10.4 was employed to combine trees, discarding 1% burn-in with a value of 100. The final tree was visualized using FigTree v1.4.4.

To cross-validate the estimated divergence times inferred from the absolute substitution rate of evolution, an alternative method utilizing fossil calibrations was employed to estimate the ages of lineage divergences using the same dataset. Previous studies indicate stem ages for Andreopsida and Tetraphidopsida at 282 and 337 mya, respectively [72]. Herein, we incorporated a normal distribution prior with a mean of 310 mya and a standard deviation of 15 mya at the root of the tree, encompassing 95% of the probability distribution between 280 and 340 mya. Additionally, we imposed constraints on the minimum age of two nodes within the phylogeny based on fossils *Meantoinea alophosioides* [73] and *Eopolytrichum antiquum* [74]. Given the morphological similarities between *Meantoinea alophosioides* and *Alophosia*, *Bartramiopsis*, and *Lyellia*, and its sister relationship with *Alophosia* in the phylogenetic position [75], a uniform prior was applied to the node of the sister clade of *Alophosia*, setting a minimum age of 132 mya and a maximum age of 310 mya. Simultaneously, a uniform prior ranging from 82.9 mya to 310 mya was implemented on the *Polytrichum* clade node, since *Eopolytrichum antiquum* was nested within the extant genus *Polytrichum* [75]. This analysis was conducted using BEAST v1.10.4 [65], as previously described.

### 4.6. Biogeographic Inference

The delineation of eight biogeographic areas was established based on paleogeographic history and plate tectonics [21,76], encompassing Asia (AS), Southeast Asia (SEA), Africa (AF), Europe (EU), North America (NA), the Neotropics (NE), Australasia (AU), and the Pacific (PA) (Figure 3). To better investigate the dispersal patterns of *P. tahitense*, the Taiwan of China was assigned to SEA in this study. This categorization is based on the observed congruence in habitat and morphological characteristics of *P. tahitense* in Taiwan with those documented in Java and Sabah. Furthermore, the European part of Russia was categorized within Asia, as the floristic origins of European Russia are more closely affiliated to Asia than Europe [76].

The reconstruction of biogeographic history was undertaken using RASP v3.2 (Reconstruct Ancestral State in Phylogenies) [77], employing two methodologies: dispersal–vicariance analysis (DIVA) [78], executed within a Bayesian framework known as S-DIVA (statistical dispersal–vicariance analysis) [79], and dispersal–extinction–cladogenesis (DEC) analysis within a likelihood framework [80,81]. To address uncertainties in phylogenetic inference and optimize the biogeographical parameters for the observed data, 10,000 MCMC trees from BEAST v1.10.4 were utilized in RASP v3.2. The initial 1000 trees were discarded as burn-in, and the subsequent 9000 trees underwent analysis using S-DIVA methods. DEC analysis was conducted on the consensus tree derived from BEAST v1.10.4 analysis. The maximum number of ancestral areas per node was unrestricted and was set at 8 for both methodologies. To prevent over-parameterization, the dispersal probabilities in the DEC model were also unconstrained.

## 5. Conclusions

Our present study described a specimen collected from Tibet, China, unequivocally identified as *P. tahitense* through a comprehensive analysis encompassing both morphological and molecular evidence. This discovery extends the known distribution range of *P. tahitense* to include Tibet, China. Our research proposes that *P. tahitense* originated during the Pleistocene epoch, approximately 1.8 million years ago, in Tibet, China, and dispersed subsequently to islands in Southeast Asia and Pacific.

## Data Availability

Data are contained in the article, including the Supplementary Material.

## Supplementary Materials

The following supporting information can be downloaded at: https://www.mdpi.com/article/10.3390/plants13060846/s1, Figure S1: 50% majority-rule consensus tree of Polytrichaceae obtained from Bayesian analysis based on *rbcL* conducted using MrBayes v3.2.7; Figure S2: 50% majority-rule consensus tree of Polytrichaceae obtained from Bayesian analysis based on *rps4* conducted using MrBayes v3.2.7; Figure S3: 50% majority-rule consensus tree of Polytrichaceae obtained from Bayesian analysis based on *trnL-F* conducted using MrBayes v3.2.7; Figure S4: 50% majority-rule consensus tree of Polytrichaceae obtained from Bayesian analysis based on *nad5* conducted using MrBayes v3.2.7; Figure S5: 50% majority-rule consensus tree of Polytrichaceae obtained from Bayesian analysis based on *ITS2* conducted using MrBayes v3.2.7; Figure S6: 50% majority-rule consensus tree of Polytrichaceae obtained from Bayesian analysis based on *rbcL*, *rps4*, *trnL-F*, *nad5* conducted using MrBayes v3.2.7; Figure S7: 50% majority-rule consensus tree of Polytrichaceae obtained from Bayesian analysis based on *rbcL*, *rps4*, *trnL-F*, *nad5*, *ITS2* conducted using MrBayes v3.2.7; Figure S8: Phylogenetic tree of Polytrichaceae obtained from maximum likelihood analysis based on *rbcL*, *rps4*, *trnL-F*, *nad5*, *ITS2* conducted using RAxML v8.2.12; Figure S9: This dated tree of Polytrichaceae was derived from the maximum clade credibility tree of Bayesian inference analysis in BEAST v1.10.4 by using absolute rate of substitution; Figure S10: This dated tree of Polytrichaceae was derived from the maximum clade credibility tree of Bayesian inference analysis in BEAST v1.10.4 by using fossil calibrations; Figure S11: Reconstruction of ancestral biogeographic areas for Polytrichaceae based on S-DIVA; Figure S12: Reconstruction of ancestral biogeographic areas for Polytrichaceae based on DEC; Table S1: List of taxa, the GenBank accession numbers and sequence sources for *rbcL*, *rps4*, *trnL-F*, *nad5*, and *ITS2* sequences in the present study.
