# Supplementary material for: A New Record of Pogonatum tahitense (Polytrichaceae) from Tibet, China: Taxonomic Description, Range Expansion, and Biogeographic History
Source: Plants (Basel). 2024 Mar 15;13(6):846. doi: 10.3390/plants13060846 (PMC10974980; doi:10.3390/plants13060846)
Supplement: Supplementary file 1 [file plants-13-00846-s001.zip › Figure S1-S12.pdf]

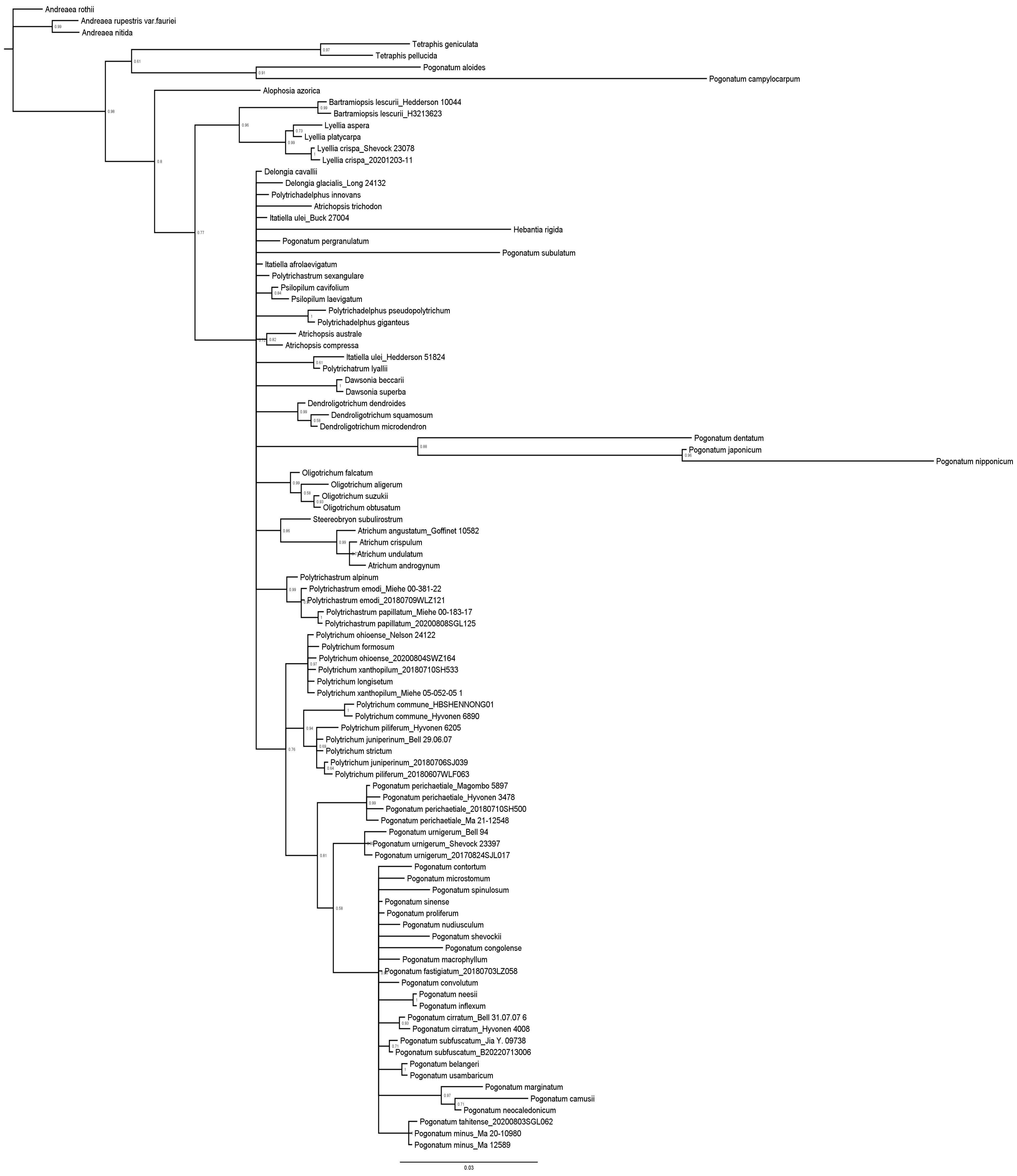

Figure S1. This 50% majority-rule consensus tree of Polytrichaceae was obtained from Bayesian analysis conducted using MrBayes v3.2.7 based on *rbcL*. Bayesian posterior probabilities are provided at each node to indicate support levels. The scale bar below the tree represents 0.03 substitutions/site.

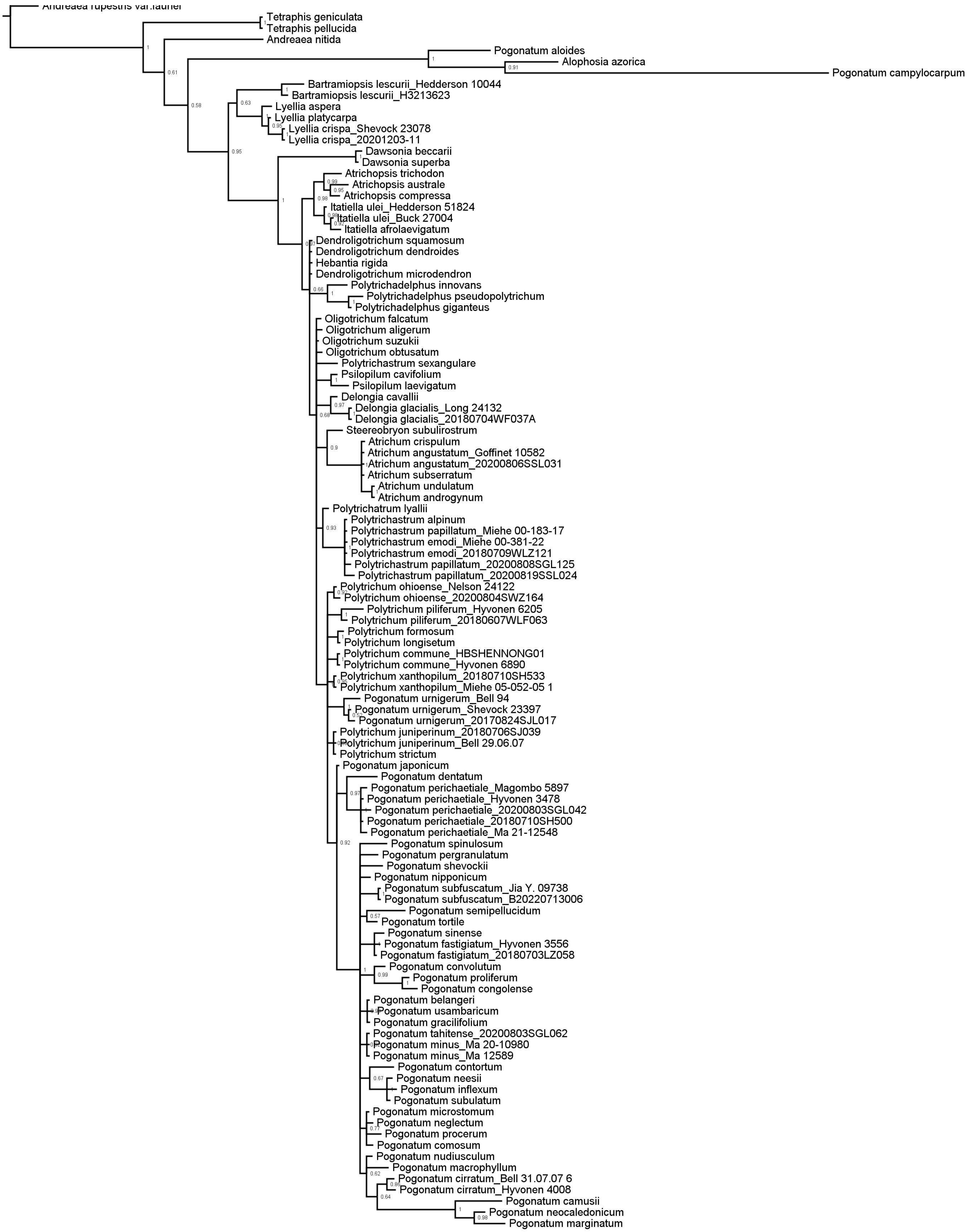

Figure S2. This 50% majority-rule consensus tree of Polytrichaceae was obtained from Bayesian analysis conducted using MrBayes v3.2.7 based on *rps4*. Bayesian posterior probabilities are provided at each node to indicate support levels. The scale bar below the tree represents 0.04 substitutions/site.

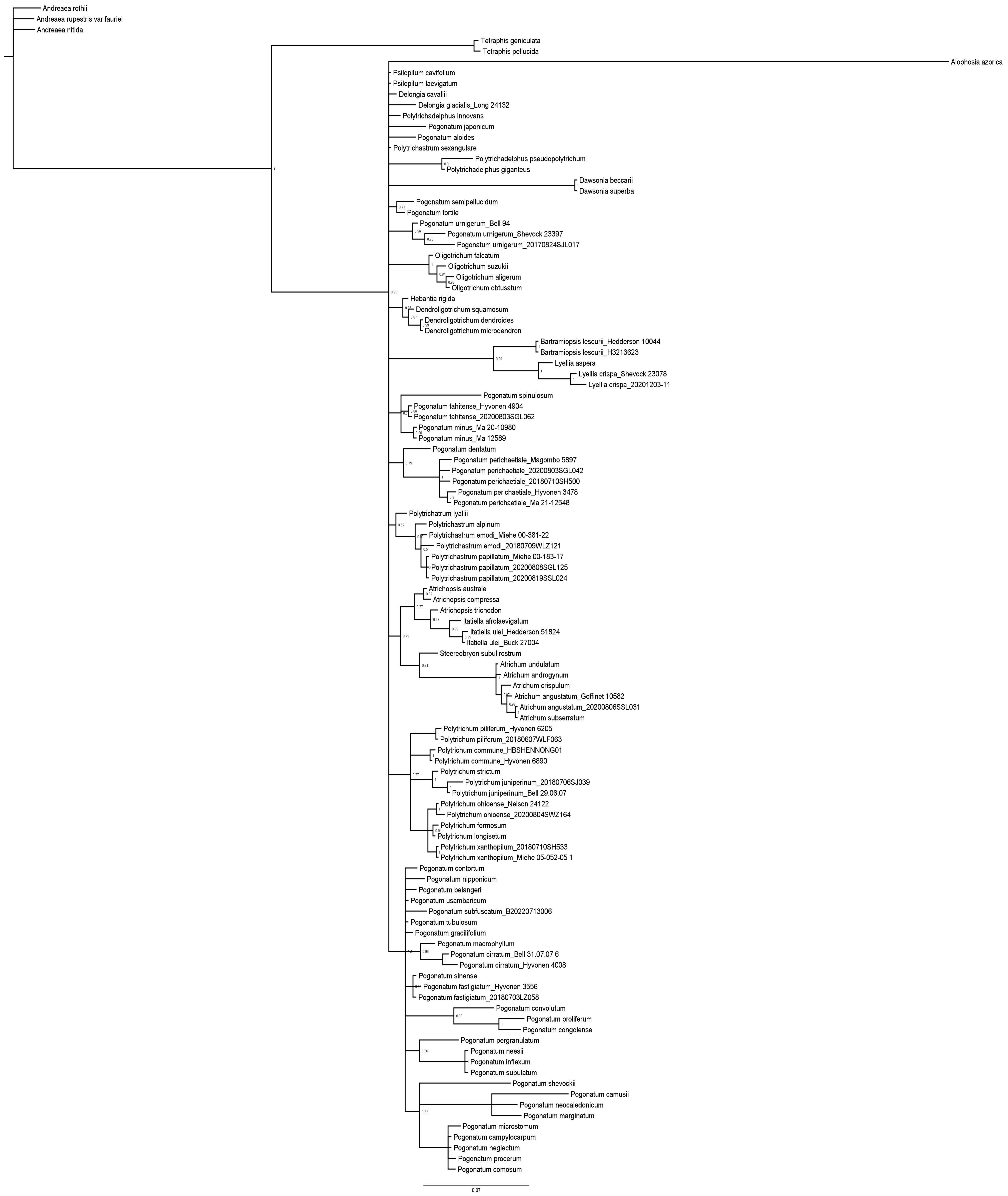

Figure S3. This 50% majority-rule consensus tree of Polytrichaceae was obtained from Bayesian analysis conducted using MrBayes v3.2.7 based on *trnL-F*. Bayesian posterior probabilities are provided at each node to indicate support levels. The scale bar below the tree represents 0.07 substitutions/site.

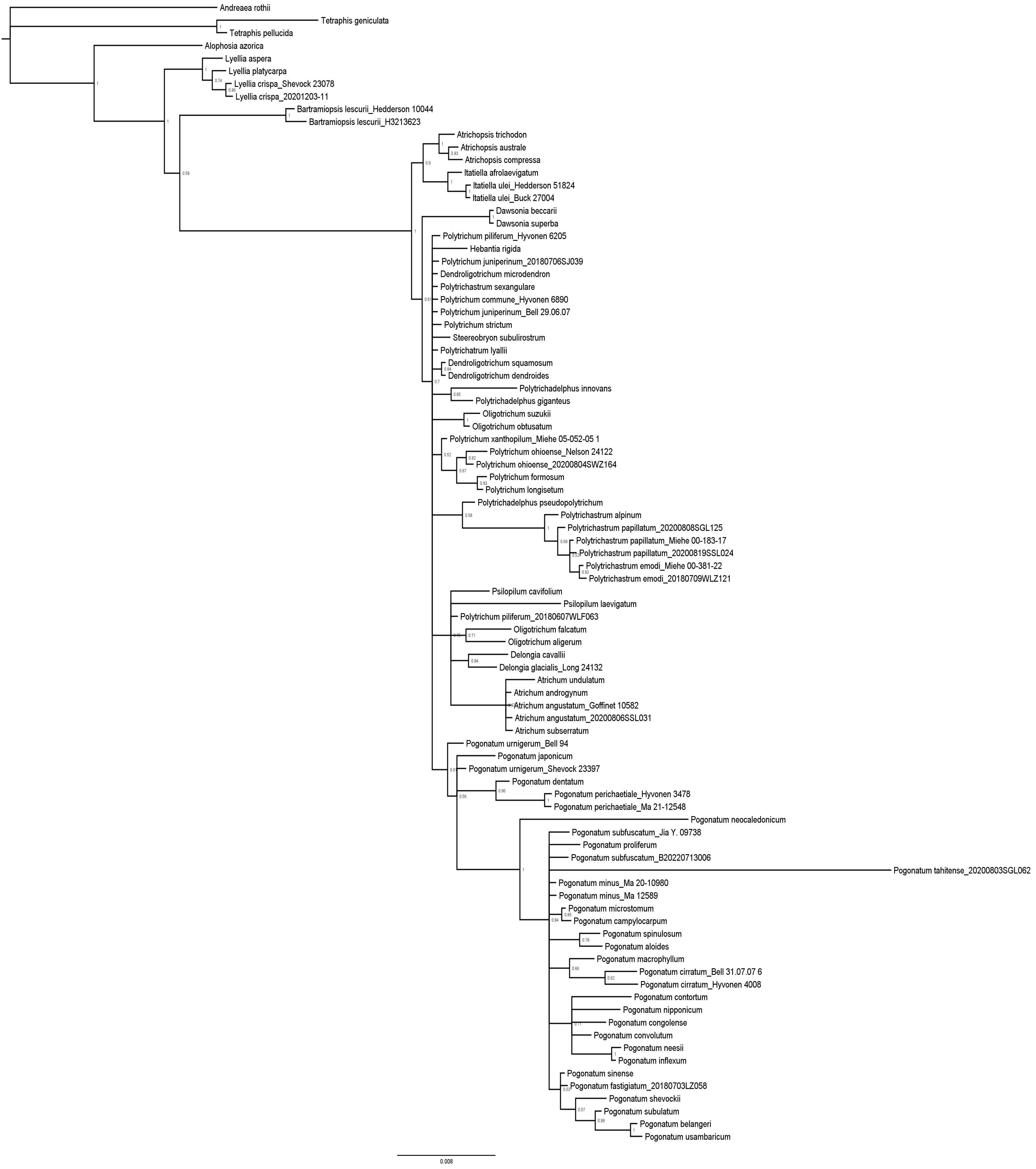

Figure S4. This 50% majority-rule consensus tree of Polytrichaceae was obtained from Bayesian analysis conducted using MrBayes v3.2.7 based on *nad5*. Bayesian posterior probabilities are provided at each node to indicate support levels. The scale bar below the tree represents 0.008 substitutions/site.

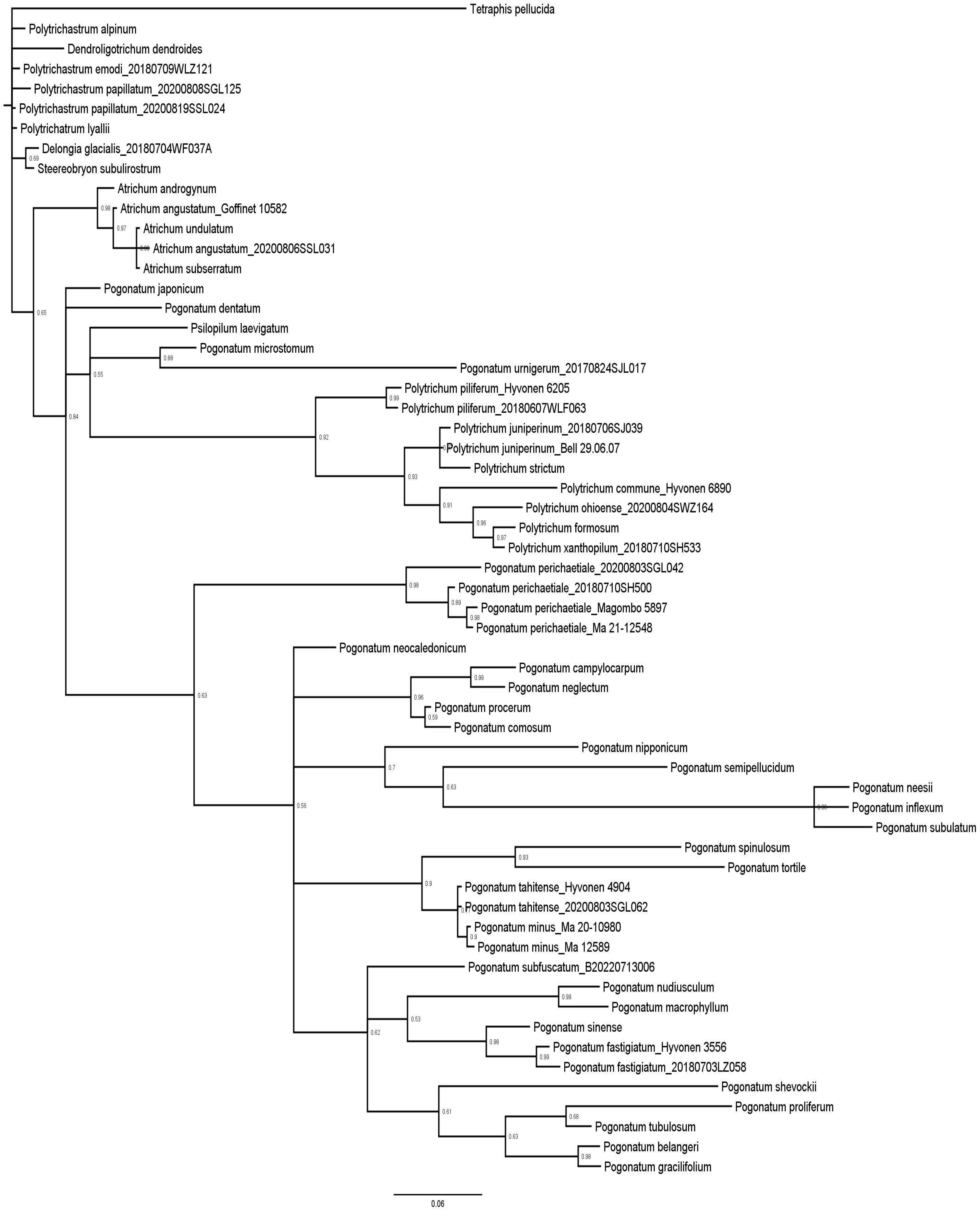

Figure S5. This 50% majority-rule consensus tree of Polytrichaceae was obtained from Bayesian analysis conducted using MrBayes v3.2.7 based on *ITS2*. Bayesian posterior probabilities are provided at each node to indicate support levels. The scale bar below the tree represents 0.06 substitutions/site.

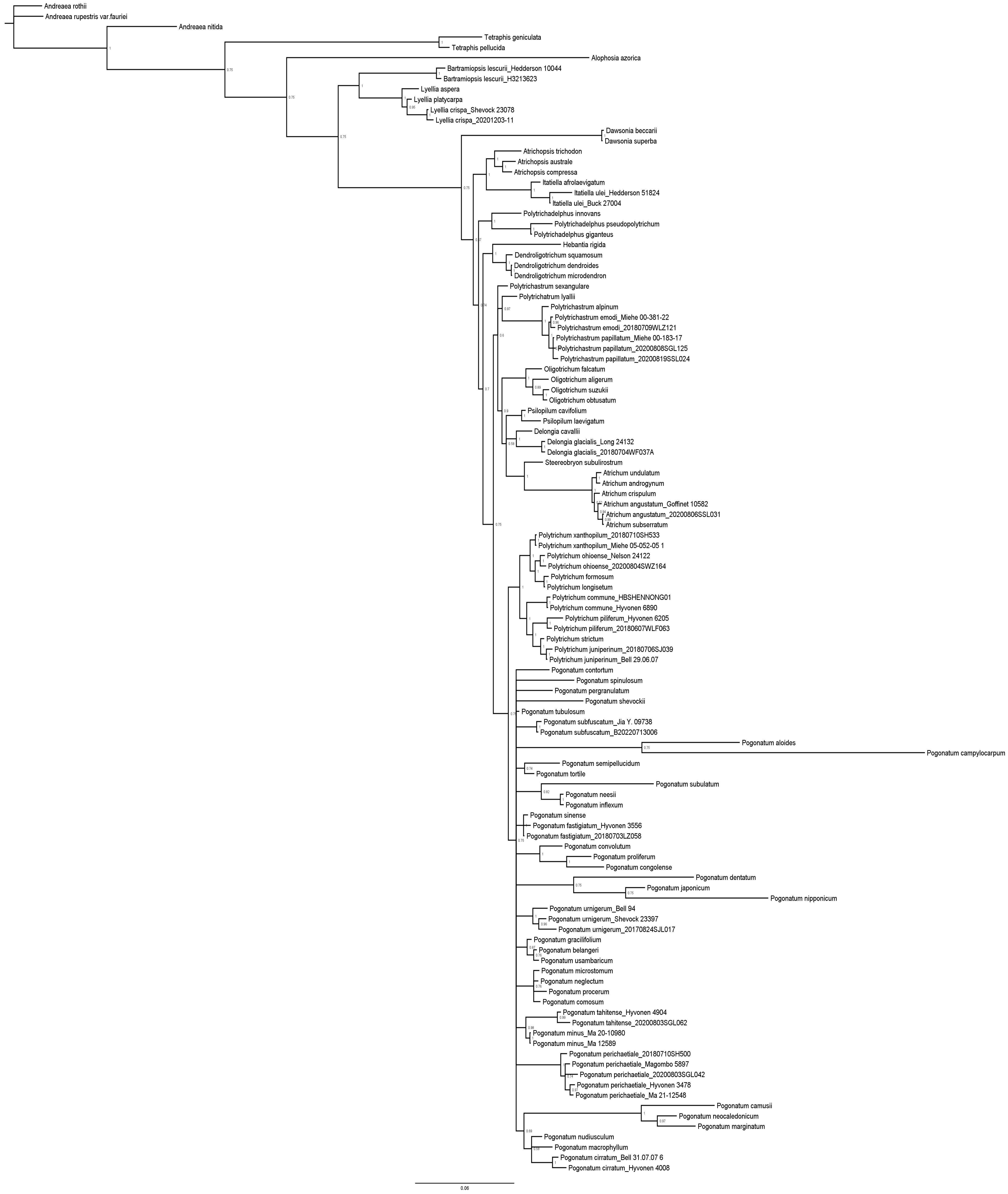

Figure S6. This 50% majority-rule consensus tree of Polytrichaceae was obtained from Bayesian analysis conducted using MrBayes v3.2.7 based on chloroplast and mitochondria regions (*rbcL*, *rps4*, *trnL-F*, *nad5*). Bayesian posterior probabilities are provided at each node to indicate support levels. The scale bar below the tree represents 0.06 substitutions/site.

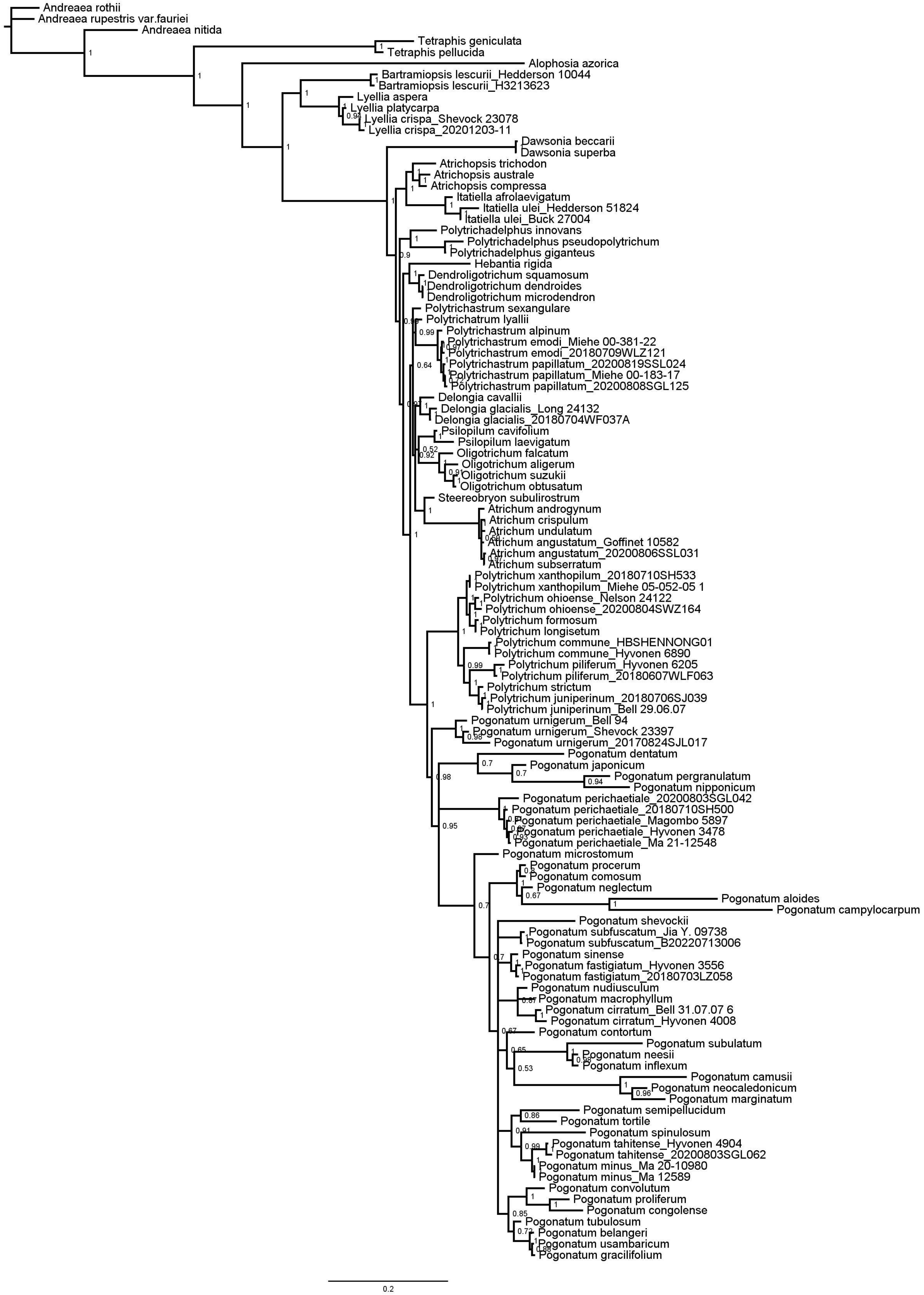

Figure S7. This 50% majority-rule consensus tree of Polytrichaceae was obtained from Bayesian analysis conducted using MrBayes v3.2.7 based on *rbcL*, *rps4*, *trnL-F*, *nad5*, and *ITS2* regions. Bayesian posterior probabilities are provided at each node to indicate support levels. The scale bar below the tree represents 0.2 substitutions/site.

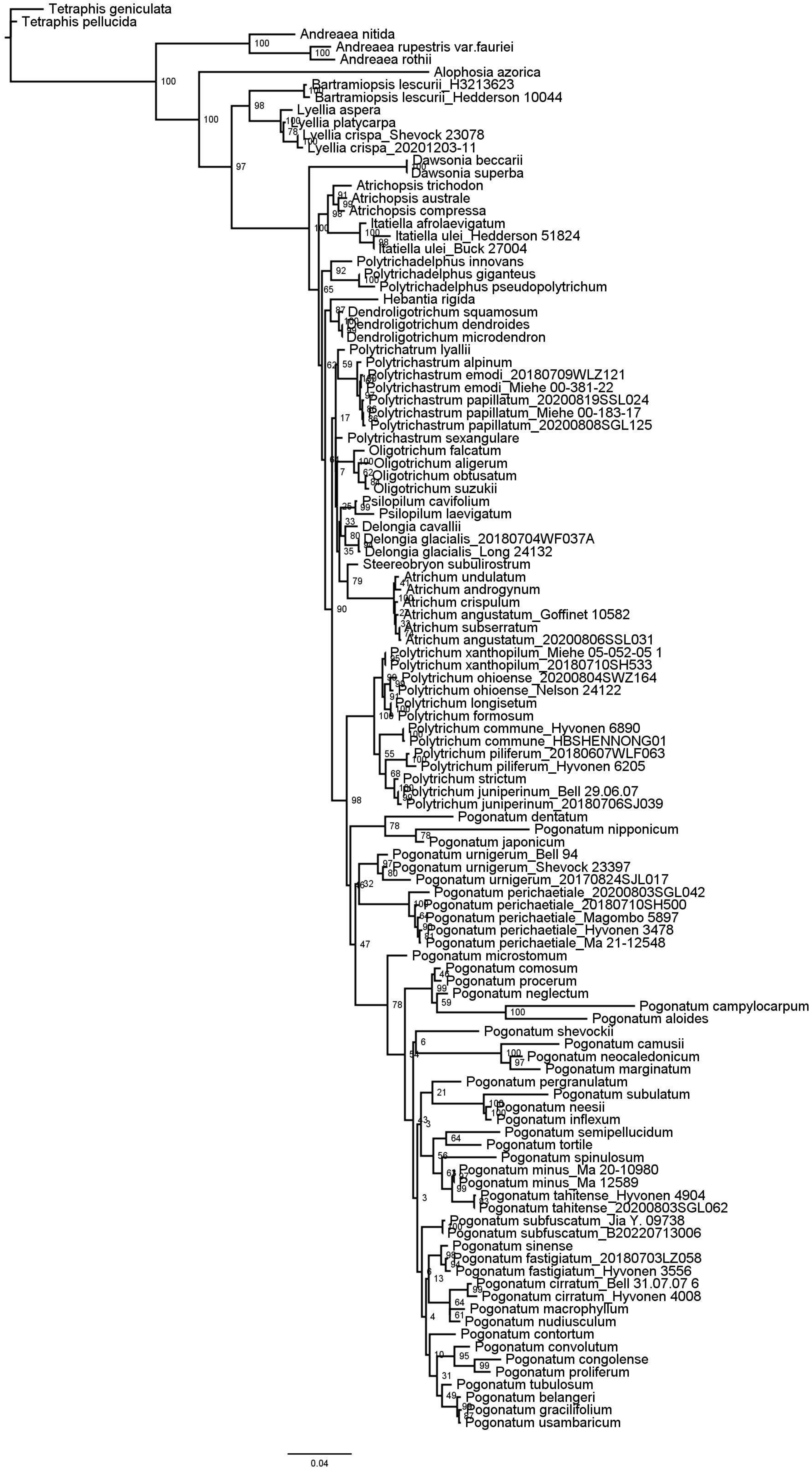

Figure S8. Phylogenetic tree of Polytrichaceae from Maximum Likelihood analysis conducted using RAxML v8.2.12 based on *rbcL*, *rps4*, *trnL-F*, *nad5*, and *ITS2*. Bootstrap values are provided at each node to indicate support levels. The scale bar below the tree represents 0.04 substitutions/site.

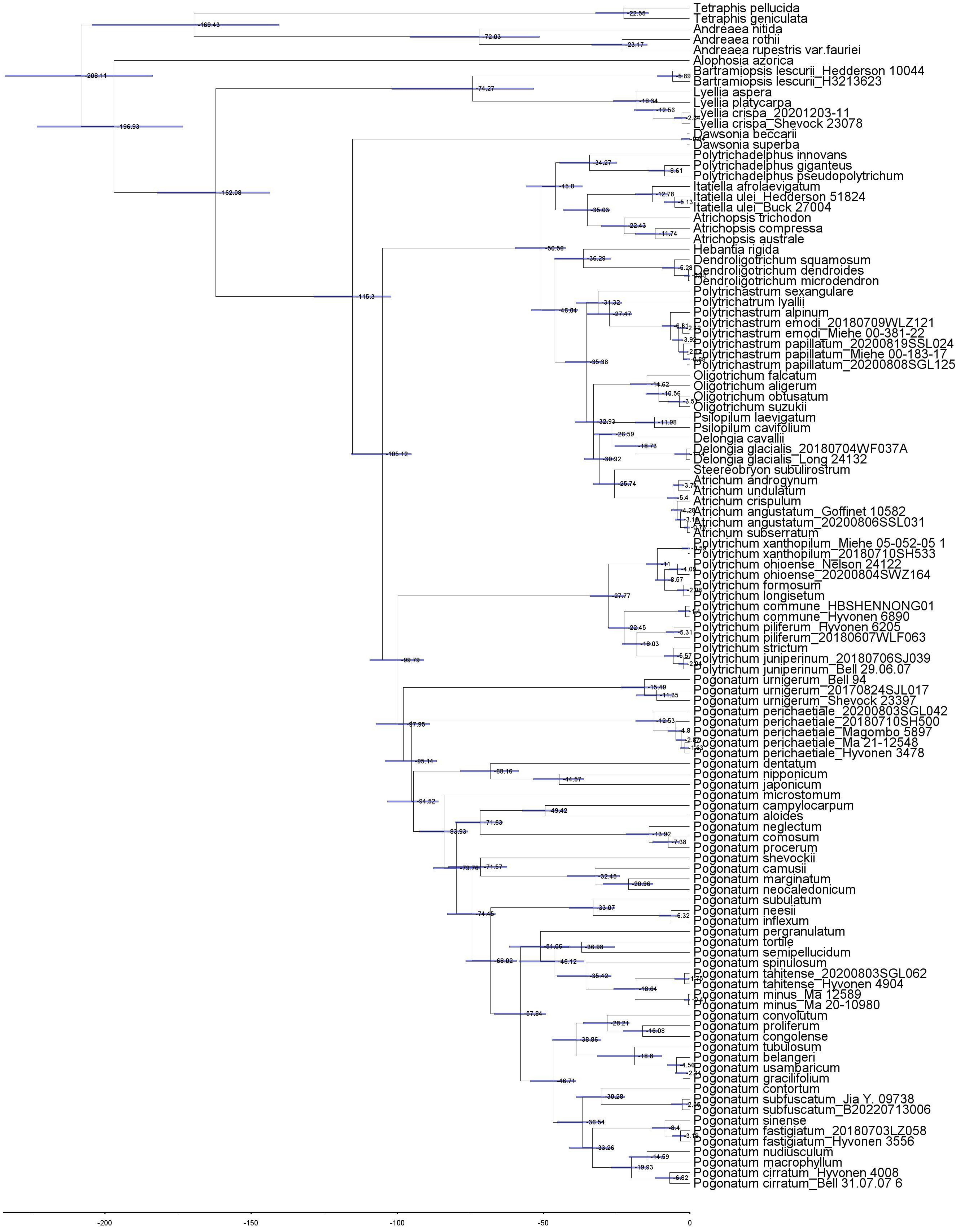

Figure S9. This dated tree of Polytrichaceae was derived from maximum clade credibility tree of Bayesian inference analysis in BEAST v1.10.4 by using absolute rate of substitution. The divergence times are given at each node. Horizontal blue bars indicate 95% probability-density interval on node age. The timescale in millions of years before present are shown below the tree.

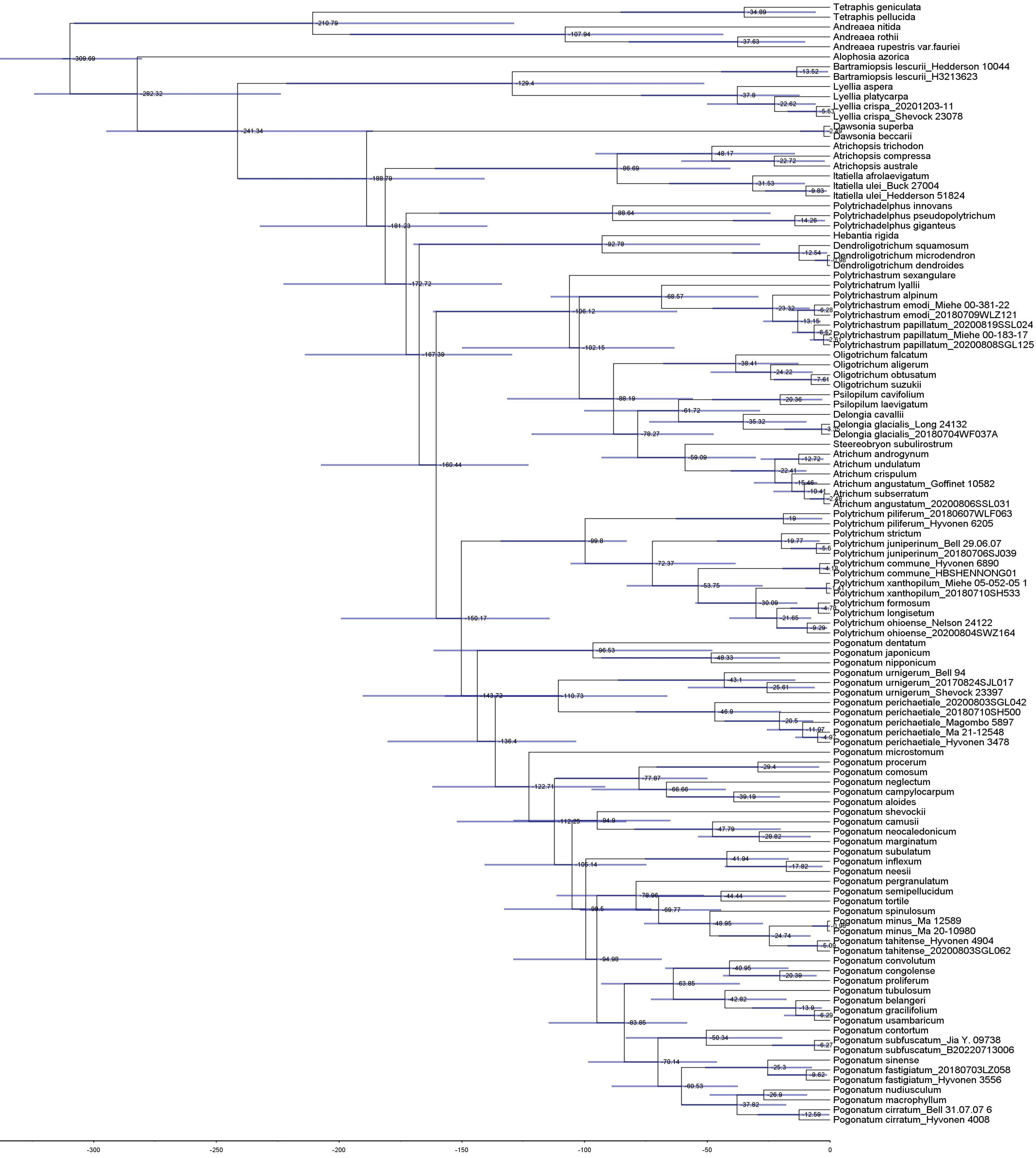

Figure S10. This dated tree of Polytrichaceae was derived from maximum clade credibility tree of Bayesian inference analysis in BEAST v1.10.4 by using fossil calibrations. The divergence times are given at each node. Horizontal blue bars indicate 95% probability-density interval on node age. The timescale in millions of years before present are shown below the tree.

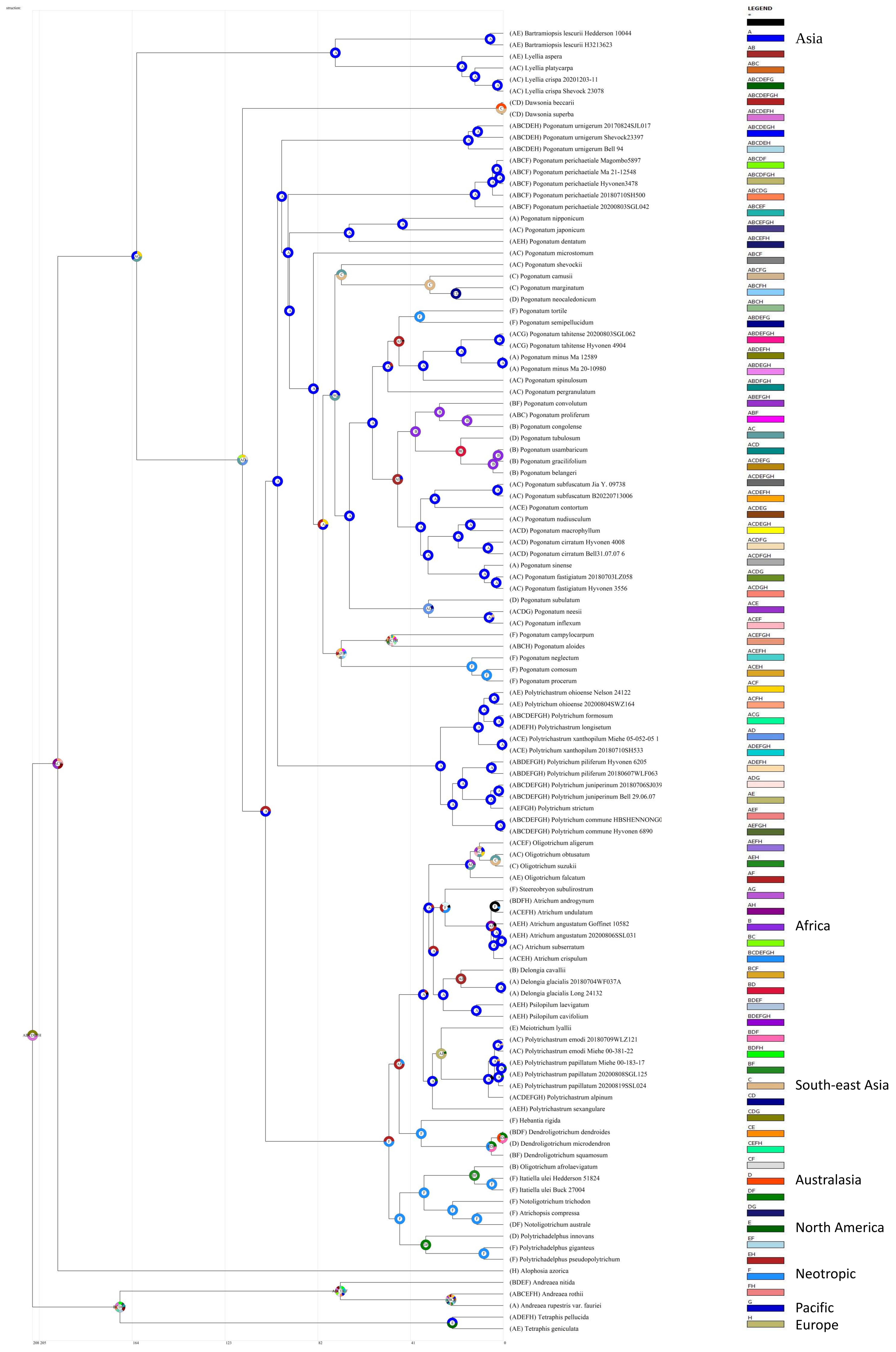

Figure S11. Reconstruction of ancestral biogeographic areas for Polytrichaceae based on statistical dispersal-vicariance analysis (S-DIVA) analysis as implemented in the software RASP v3.2. Colored circles at each node indicate the potential ancestral area, the most likely ancestral area is shown in the center. The timescale in millions of years before present are shown below the tree. Asterisk indicates the specimen collected from Tibet, China.

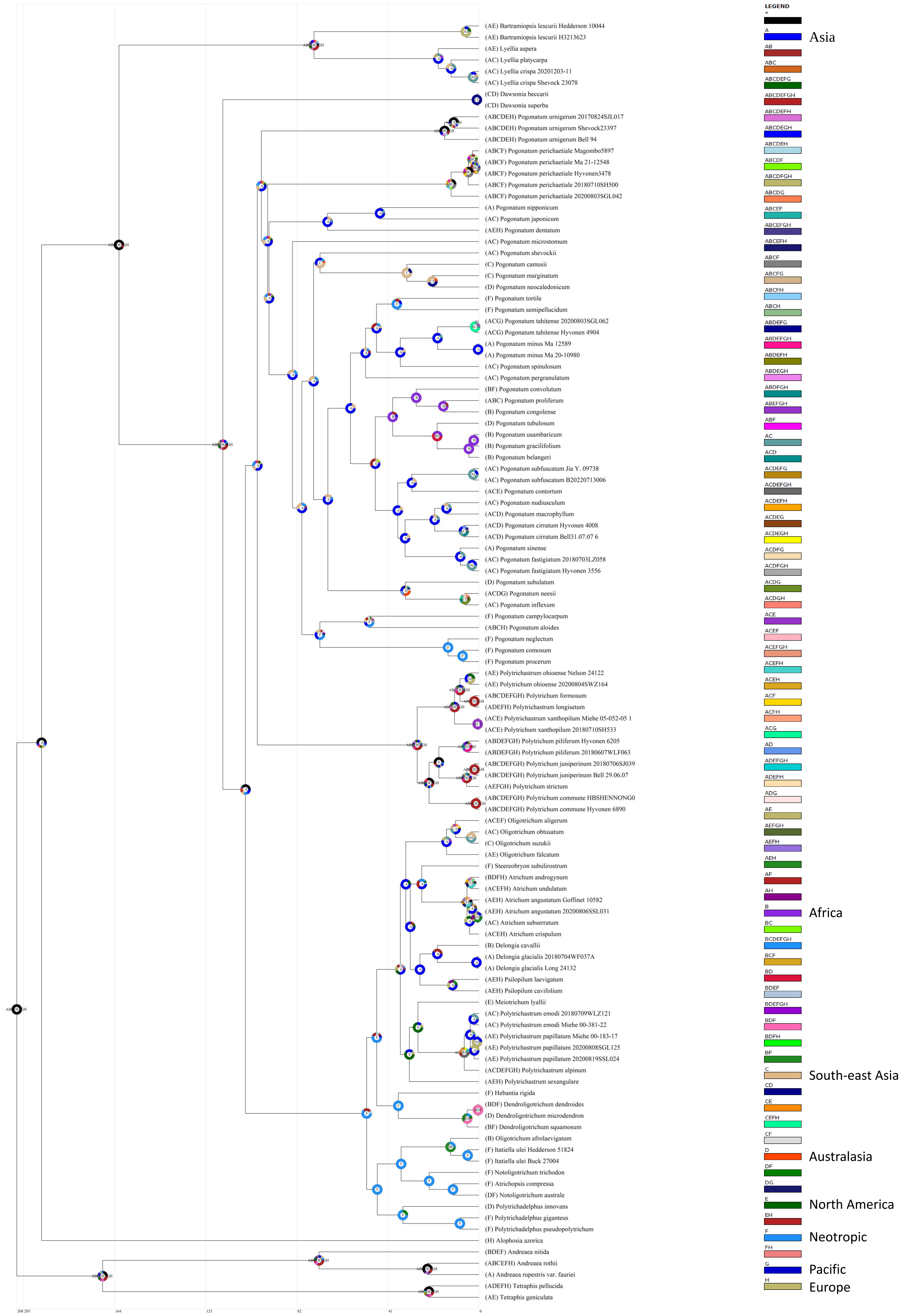

Figure S12. Reconstruction of ancestral biogeographic areas for Polytrichaceae based on dispersal-extinction-cladogenesis (DEC) analysis as implemented in the software RASP v3.2. Colored circles at each node indicate the potential ancestral area, the most likely ancestral area is shown in the center. The timescale in millions of years before present are shown below the tree. Asterisk indicates the specimen collected from Tibet, China.
