# Supplementary material for: A New Record of Pogonatum tahitense (Polytrichaceae) from Tibet, China: Taxonomic Description, Range Expansion, and Biogeographic History
Source: Plants (Basel). 2024 Mar 15;13(6):846. doi: 10.3390/plants13060846 (PMC10974980; doi:10.3390/plants13060846)
Supplement: Supplementary file 1 [file plants-13-00846-s001.zip › Table S1.pdf]

Table S1. List of taxa, the GenBank accession numbers and sequence sources for *rbcL*, *rps4*, *trnL-F*, *nad5* and *ITS2* sequences in the present study.

Taxon	Genbank accession numbers					Voucher or sequence sources
	rbcL	*rps4*	*trnL-F*	*nad5*	*ITS2*	
Alophosia azorica	AF208408	AY137679	AF544997	AY137713	AY330424	Rumsey 18.3.1997
Andreaea nitida	AF478198	AF478247	AF478293	/	/	Churchill et al. 19834, MO
Andreaea rothii	AY608025	AY312866	AY608120	AY312862	/	Shaw 11565 (DUKE)
Andreaea rupestris var. *fauriei*	FJ572340		FJ572421	/	/	Liu et al., 2010
		MW561627				Zhu & Zhang 20200723-14
Atrichopsis australe	AF208414	AY137686	AF545012	AY137727	/	Hyvonen 6069
Atrichopsis compressa	EU927307	EU927332	GU569669	GU569492	/	Bell 1615, H
Atrichopsis trichodon	GU569428	GU569782	GU569694	GU569517	/	Bell 1493, H
Atrichum androgynum	AY118234	GU569763	AF544999	AY137714	AY396432	Hyvonen 6387
Atrichum angustatum	/	PP313605	PP001229	PP313609	PP291841	20200806SSL031
Atrichum angustatum	NC_058541	KC663272				Goffinet 10582
			AF545000	AY137715	AY396430	Hedderson 10393
Atrichum crispulum	KP881776	KP881849	KP881811	/	/	Perley & Jesson 2015
Atrichum subserratum	/	PP001222	PP313608	PP313616	PP035987	20200804SGL081
Atrichum undulatum	AY118236	AY137681	AF545002		AY396433	Hyvonen 6170
				AJ001229		Beckert 1999
Bartramiopsis lescurii	AF208409	AF208418	AF545003	AY137718	/	Hedderson 10044
Bartramiopsis lescurii	MZ368539	MZ368541	MZ368544	MZ368547	/	H3213623 I.V. Chernyadjeva
Dawsonia beccarii	GU569412	GU569766	GU569674	GU569497	/	H:Bell 31.07.07 #35
Dawsonia superba	GU569413	GU569767	GU569675	GU569498	/	H:Bell 31.07.07 #24
Delongia cavallii	JQ639452	JQ639424	JQ639414		/	Porely U9150a, H
				KP901298		BOL:Hedderson 17887
Delongia glacialis	KP901282	KP901288	KP901294	KP901300	/	Long 24132
Delongia glacialis	/	PP001218	/	/	PP035985	20180704WF037A
Dendroligotrichum dendroides	AF208420	AF208411	AF545006	AY137721		Hyvonen 6083
					AY028983	Pfeiffer，Unpublished
Dendroligotrichum microdendron	GU569414	GU569768	GU569677	GU569500	/	H:Bell 29.02.08 #2
Dendroligotrichum squamosum	AY118239	AY137684	AF545007	AY137722	/	Hyvonen 2557
Hebantia rigida	AY118240	AY137685	AF545008	AY137723	/	Kelt 26.5.1986
Itatiella afrolaevigatum	GU569429	GU569783	GU569695	GU569518	/	H:Magill 4340
Itatiella ulei	GU569418	GU569772	GU569681	GU569504	/	Buck 27004
Itatiella ulei	AF208412	AF208421	AF545009	GU569503	/	Hedderson 51824
Lyellia aspera	GU569419	GU569773	GU569682	GU569505	/	H:Afonina 8/3, 10.07.06
Lyellia crispa	PP001227	PP001221	PP313607	PP313615	/	20201203-11

Lyellia crispa	EU927310	EU927335	GU569683	GU569506	/	Shevock 23078
Lyellia platycarpa	JX241623	JX241479	/	JX241612	/	PE:Jia Y. 09917
Oligotrichum aligerum	JQ639451	JQ639423	JQ639413	JQ639433	/	Norris 83978, H
Oligotrichum falcatum	JQ639453	JQ639426	JQ639416	JQ639435	/	Ignatov 97-525, H
Oligotrichum obtusatum	GU569433	GU569787	GU569699	GU569522	/	H:Hyvonen 3469
Oligotrichum suzukii	GU569435	GU569789	GU569701	GU569524	/	H:Hyvonen 3985
Pogonatum aloides	AY118244	AY137689	AF545016		/	Hyvonen 6486
				GU569526		H:Bell 04.01.07 #1
Pogonatum belangeri	GU569438	GU569792	GU569704	GU569527		H:Hedderson 16289
					AY396474	Laaka 298
Pogonatum brachyphyllum	/	AY396515	AY396497		AY396473	Shaw 8304
				AY908806		Buck 31598 (NY)
Pogonatum campylocarpum	AY118245	AY137690	AF545017	GU569528	AY396455	H:Hyvonen 06392
Pogonatum camusii	KU852692	KU852695	KU852698	/	/	H:Menzel 4266
Pogonatum cirratum	GU569440	GU569794	GU569706	GU569529	/	Bell 31.07.07 6
Pogonatum cirratum	AY118246	AY137691	AF545018	AY137733	/	Hyvonen 4008
Pogonatum comosum	/	AY396503	AY396481	/	AY396446	Norris 77508
Pogonatum congolense	KY793620	KY793654	KY793629	KY793647	/	H:Shevock 40161
Pogonatum contortum	AY118247	AF208425	AF545019	AY137734	/	Hedderson 5803
Pogonatum convolutum	GU569442	GU569796	GU569708	GU569531	/	H:Hedderson 16265
Pogonatum dentatum	AY118248	AY137692	AF545020	AY137735	AY396454	Hyvonen 6169
Pogonatum fastigiatum	PP001244	PP001207	PP001230	/	PP035975	20180703LZ058
Pogonatum fastigiatum	/	AY396504	AY396482	to be updated	AY396447	Hyvonen 3556
Pogonatum gracilifolium	/	AY396507	AY396488	/	AY396462	Wigginton 5071a
Pogonatum inflexum	MK131349	MK131349		MK131350		Zheng & Meng, unpublished
			AY396486		AY396459	Chishiki 1865
Pogonatum japonicum	AY118249	GU569798	GU569710	GU569533	AY396463	H:Nishimura 10601
Pogonatum macrophyllum	GU569445	GU569799	GU569711	GU569534		H:Bell 30.07.07 #19
					AY396443	Tan s.n.
Pogonatum marginatum	KU852693	KU852696	KU852699	/	/	H:He 1485
Pogonatum microstomum	GU569446	GU569800	GU569712	GU569535		H:Shevock 22895
					AY396450	Hyvonen 4087
Pogonatum minus	PP059838	PP034674	PP034677	PP034680	PP038302	Ma 20-10980
Pogonatum minus	PP059840	PP034676	PP034679	PP034682	PP038304	Ma 12589
Pogonatum nanum	/	AY396506	AY396484	/	AY396456	Hyvonen 6484
Pogonatum neesii	AY118251	AY137695	AF545023	AY137738	AY396449	Hyvonen 4021
Pogonatum neglectum	/	AY396510	AY396491	/	AY396466	Churchill 16370
Pogonatum neocaledonicum		AY396513	AY396495		AY396471	Norris 93319

	KY793623			KY793648		H:Bell 07.11.08.007
Pogonatum nipponicum	AY118252	AY137696	AF545024	GU569537	AY396460	H:Hayashi 7038
Pogonatum nudiusculum	MT324552	AY396505	/	/	AY396451	Hyvonen 4153
Pogonatum pensilvanicum	AY118253	/	AF545025	AY137740		Hyvonen 6393
					AY396442	Goffinet 5266
Pogonatum pergranulatum	/	AY396514	AY396496	/	/	Allen 6501
Pogonatum perichaetiale	/	PP001209	PP001232	/	PP035977	20200803SGL042
Pogonatum perichaetiale	PP001226	PP001217	PP001240	/	PP035984	20180710SH500
Pogonatum perichaetiale	PP059839	PP034675	PP034678	PP034681	PP038303	Ma 21-12548
Pogonatum perichaetiale	GU569449	GU569803	GU569715	GU569538	/	Hyvonen 3478
Pogonatum perichaetiale	AF478206	AF478258				Magombo 5897
			AY396487	/	AY396461	Lewis 87019 (MO)
Pogonatum procerum	/	AY396508	AY396489	/	AY396464	Liesner 26480
Pogonatum proliferum	GU569450	GU569804	GU569716	GU569539		H:Bell 31.07.07 #9
					AY396465	Porley U652a
Pogonatum semipellucidum	/	AY396511	AY396492	/	AY396467	Churchill 18679
Pogonatum shevockii	KY793627	KY793661	KY793636	KY793652	KY793640	E:Shevock & Yao 47925
Pogonatum sinense	KP901283	KP901289	KP901295	KP901301		E:Long 24276
					AY396445	Long 8696
Pogonatum spinulosum	AY118254	AY137698	AF545026	AY137741	AY396457	Chishiki 1862
Pogonatum subfuscatum	PP001224	PP001214	PP001237	PP001246	PP035981	B20220713006
Pogonatum subfuscatum	JX241624	JX241476	/	JX241613	/	Jia Y. 09738
Pogonatum subulatum	AY118255	AY137699	AF545027	AY137742	AY396453	Hyvonen 6025
Pogonatum tahitense	PP313604	PP001220	PP001242	PP001248	PP035986	20200803SGL062
Pogonatum tahitense	/	/	AY396483	/	AY396452	Hyvonen 4904
Pogonatum tortile	/	AY396501	AY396476	/	AY396441	Goffinet 5067
Pogonatum tubulosum	/	/	AY396485	/	AY396458	Hoffmann 89-741
Pogonatum urnigerum	PP313603	PP001219	PP001241	/	PP048943	20170824SJL017
Pogonatum urnigerum	GU569453	GU569807	GU569719	GU569542	/	H:Bell 01.08.07 #94
Pogonatum urnigerum	GU569454	GU569808	GU569720	GU569543	/	H:Shevock 23397
Pogonatum usambaricum	GU569455	GU569809	GU569721	GU569544		H:Hedderson 16241
					AY396475	Laaka 3235
Polytrichadelphus giganteus	GU569457	GU569811	GU569723	GU569546	/	H:Churchill & Betancur 18057
Polytrichadelphus innovans	GU569458	GU569812	GU569725	GU569548	/	H:Bell 04.03.08 #4
Polytrichadelphus pseudopolytrichum	AF261074	AY137700	AF545030	AY137745	/	Hyvoenen 6276
Polytrichastrum alpinum	GU569464	GU569818	GU569733	GU569556		H:Bell 02.07.06 #3
					MF180534	Biersma et al., 2017
Polytrichastrum emodi	PP001223	PP001210	PP001233	PP313611	PP035978	20180709WLZ121

Polytrichastrum emodi	GU569467	GU569821	GU569736	GU569559	/	Miehe 00-381-22
Polytrichastrum papillatum	PP001225	PP001215	PP001238	PP001247	PP035982	20200808SGL125
Polytrichastrum papillatum	/	PP001216	PP001239	PP313614	PP035983	20200819SSL024
Polytrichastrum papillatum	GU569474	GU569828	GU569744	GU569567	/	Miehe 00-183-17
Polytrichastrum sexangulare	GU569476	GU569830	GU569746	GU569569	/	H:Belland 5727
Polytrichatrum lyallii	AY118241	AF208423	AF545011	AY137726	AY396426	Weber 36612
Polytrichum commune	PP313600	PP001211	PP001234	/	/	HBSHENNONG01
Polytrichum commune	GU569483	GU569837	GU569753	GU569576		Hyvonen 6890
					AY396437	Hyvonen 6168
Polytrichum formosum	AY118259	AY137702	AF545032	AY137747	AY396435	Hyvonen 6197
Polytrichum juniperinum	PP313601	PP001212	PP001235	PP313612	PP035979	20180706SJ039
Polytrichum juniperinum	EU927317	EU927342	GU569757	GU569581		Bell 29.06.07
					EF590796	NMNH 33767
Polytrichum longisetum	AY118260	AY137703	AF545033	AY137748	/	Hyvonen 6506
Polytrichum ohioense	PP001245	PP001208	PP001231	PP313610	PP035976	20200804SWZ164
Polytrichum ohioense	GU569472	GU569826	GU569742	GU569565	/	Nelson 24122
Polytrichum piliferum	PP313602	PP001213	PP001236	PP313613	PP035980	20180607WLF063
Polytrichum piliferum	AY118263	AY137706	AF545037	AY137752	AY396439	Hyvonen 6205
Polytrichum strictum	GU569489	GU569842	GU569759	GU569583		H:Bell 1775
					MF180503	AAS:700a
Polytrichum xanthopilum	PP001228	PP313606	PP001243	/	PP291840	20180710SH533
Polytrichum xanthopilum	GU569482	GU569836	GU569752	GU569575	/	Miehe 05-052-05 1
Psilopilum cavifolium	EU927318	EU927343	GU569761	GU569585	/	H:Bell 05.07.06 #2
Psilopilum laevigatum	AF208416	AF208429	AF545039	AY137754	AY396434	Hedderson 5938
Steereobryon subulirostrum	AY118265	AY137708	AF545040	AY137755	AY396429	Hedderson 12898
Tetraphis geniculata	AY118232	AF306955	AF544996	AY137712	/	Schofield 103022 (DUKE)
Tetraphis pellucida	U87091	AF231896	AF231908	AJ224855		Pittillo 9764(DUKE)
					FJ572397	Liu et al., 2010
